# Double-Masked, Randomized, Phase 2 Evaluation of Abicipar Pegol (an Anti-VEGF DARPin Therapeutic) in Neovascular Age-Related Macular Degeneration

**DOI:** 10.1089/jop.2018.0062

**Published:** 2018-12-06

**Authors:** David Callanan, Derek Kunimoto, Raj K. Maturi, Sunil S. Patel, Giovanni Staurenghi, Sebastian Wolf, Janet K. Cheetham, Thomas C. Hohman, Kimmie Kim, Francisco J. López, Susan Schneider

**Affiliations:** ^1^ Texas Retina Associates, Arlington, Texas.; ^2^ Retinal Consultants of Arizona, Phoenix, Arizona.; ^3^ Midwest Eye Institute, Indianapolis, Indiana.; ^4^ Department of Ophthalmology, Indiana University School of Medicine, Indianapolis, Indiana.; ^5^ West Texas Retina, Abilene, Texas.; ^6^ Department of Biomedical and Clinical Sciences, Eye Clinic, University of Milan, “Luigi Sacco” Hospital, Milan, Italy.; ^7^ Department of Ophthalmology and Bern Photographic Reading Center, Inselspital, Bern University Hospital, University of Bern, Bern, Switzerland.; ^8^ Allergan plc, Irvine, California.

**Keywords:** abicipar pegol, age-related macular degeneration, anti-VEGF, choroidal neovascularization, optical coherence tomography, vascular endothelial growth factor

## Abstract

***Purpose:*** To evaluate safety and efficacy of the vascular endothelial growth factor binding protein abicipar pegol (abicipar) versus ranibizumab for neovascular age-related macular degeneration.

***Methods:*** Phase 2, multicenter, randomized, double-masked comparison (REACH study, stage 3). Patients (*n* = 64) received intravitreal injections of abicipar 1 mg or 2 mg at baseline, week 4, and week 8 (3 injections) or ranibizumab 0.5 mg at baseline and monthly (5 injections).

***Results:*** In the abicipar 1 mg (*n* = 25), abicipar 2 mg (*n* = 23), and ranibizumab (*n* = 16) arms, respectively, least-squares mean best-corrected visual acuity (BCVA) change from baseline was +6.2, +8.3, and +5.6 letters at week 16 (primary endpoint) and +8.2, +10.0, and +5.3 letters at week 20. Least-squares mean central retinal thickness (CRT) reduction from baseline was 134, 113, and 131 μm at week 16 and 116, 103, and 138 μm at week 20. Intraocular inflammation adverse events (AEs), reported in 5/48 (10.4%) abicipar-treated patients, resolved without sustained vision loss or other sequelae.

***Conclusions:*** Abicipar demonstrated durability of effect: BCVA and CRT improvements were similar between abicipar and ranibizumab at weeks 16 and 20 (8 and 12 weeks after the last abicipar injection and 4 weeks after the last ranibizumab injection). No serious AEs were reported.

## Introduction

Age-related macular degeneration (AMD), characterized by progressive degeneration of photoreceptors and retinal pigment epithelium (RPE), is the leading cause of irreversible blindness in older individuals residing in developed countries.^1^ It is estimated that approximately 9 million people in the United States had large drusen or advanced stage AMD in 2000, and this number is projected to almost double by the year 2020.^2^ The disease is typically classified as early AMD when medium drusen (≥63 to ≤125 μm) are present and intermediate AMD when large drusen (>125 μm), or medium drusen associated with pigmented abnormalities, are present.^3^ The advanced forms of AMD are characterized by geographic atrophy or neovascularization.^1,3^ In neovascular AMD (nAMD), newly formed immature blood vessels, originating from the choroid, break through Bruch's membrane and extend below the RPE or invade the subretinal space. Choroidal neovascularization (CNV) and fluid exudation from the immature vessels are associated with tissue disruption, subretinal disciform scar formation with subsequent atrophy of the adjacent neurosensory retina, and much of the severe vision loss that occurs in patients with AMD.^1^

Characterization of the endothelial cell proliferative and vascular permeability effects of vascular endothelial growth factor (VEGF) led to the development of anti-VEGF treatments for nAMD. The registration studies with the anti-VEGF antibody fragment ranibizumab (Lucentis^®^, Genentech, South San Francisco, CA) demonstrated inhibition of CNV lesion growth and exudation and improvement in best-corrected visual acuity (BCVA) in the eyes of patients with nAMD when ranibizumab was administered on a fixed monthly treatment schedule.^4,5^ Retreatment criteria largely based on BCVA and central retinal thickness (CRT) were adopted in the PrONTO and SUSTAIN studies in an attempt to reduce monthly intravitreal injection burden, while maintaining efficacy.^6,7^ Results from the 40-patient PrONTO study were encouraging, with a mean BCVA improvement of 9.3 letters in patients receiving an average of 5.6 intravitreal injections over a 12-month period.^6^ In the 513-patient SUSTAIN study, which had similar inclusion/exclusion criteria and baseline lesion characteristics, the mean improvement in BCVA at month 12 was only 3.6 letters, with an average of 5.6 intravitreal injections over the 12-month study.^7^ Similarly, results from the HORIZON trial suggested that less frequent ranibizumab treatment results in an incremental decline in BCVA from that achieved with monthly treatment.^8^ These studies emphasized the need for therapies that provide efficacy equivalent to monthly intravitreal ranibizumab, but with a reduced number of injections.

DARPin^®^ therapeutics (a registered trademark of Molecular Partners AG, Switzerland; DARPin was originally derived from designed ankyrin repeat protein) represent a novel class of protein binding molecules under evaluation as potential therapeutics for a number of indications in oncology and ophthalmology, and for human immunodeficiency virus and allergic reactions including allergic asthma.^9–11^ Abicipar pegol (AGN-150998, MP0112, abicipar; Allergan plc/Molecular Partners) is a member of this class of protein binding molecules that specifically binds with high affinity to all soluble isoforms of VEGF-A.^12^ Abicipar has a smaller molecular weight (34 kDa vs. 48 kDa),^12^ higher target binding affinity (2 pM vs. 46 pM),^13,14^ and longer ocular half-life (half-life of ≥13 days vs. 7 days in the aqueous humor) than ranibizumab.^13,15^ These properties are believed to contribute to greater durability of action compared with currently available anti-VEGF therapies.

Abicipar was evaluated in a phase 2 study (REACH) in patients with nAMD. REACH was conducted in 3 stages. Stage 1 evaluated the safety of abicipar after a single intravitreal injection in patients with advanced nAMD (*n* = 24). Stage 2 assessed the safety and treatment effects of abicipar compared with ranibizumab using as-needed administration in treatment-naive nAMD patients (*n* = 183). Based on stage 1 and 2 outcomes, stage 3 (*n* = 64) was designed to directly compare the efficacy, safety, and systemic pharmacokinetic profile of 1-mg and 2-mg injections of abicipar compared with ranibizumab 0.5 mg for the treatment of nAMD. In addition, durability of abicipar effects was assessed for potential dosing intervals of 8 and 12 weeks. REACH stage 3 is the focus of this article.

## Methods

The phase 2 REACH stage 3 study was a 20-week, multicenter, randomized, parallel-group, double-masked comparison of the safety and treatment effects of repeat abicipar and ranibizumab administration on BCVA and retinal edema in treatment-naive patients with nAMD. REACH stage 3 was conducted from September 29, 2011 to April 9, 2014. The REACH stage 3 protocol adhered to the tenets of the Declaration of Helsinki and was approved by an institutional review board or ethics committee for each clinical site, and all participants provided written informed consent. The REACH study is registered with the identifier NCT01397409 at ClinicalTrials.gov.

### Study population

Inclusion criteria included the presence of active CNV secondary to AMD in the study eye, defined as subfoveal or juxtafoveal lesions (within 200 μm of the center of the foveal avascular zone) with leakage affecting the fovea, as confirmed by the central reading center (CRC; Bern Photographic Reading Center, Bern, Switzerland). The CNV must have been diagnosed within the previous 12 months. BCVA in the study eye was required to be between 75 and 24 letters (∼20/32 and 20/320 Snellen equivalent). Active CNV was defined by fluorescein leakage on fluorescein angiography and/or the presence of retinal fluid within or below the retina or below the RPE, as assessed with spectral-domain optical coherence tomography (SD-OCT). The area of CNV within the lesion was required to be >50% of the total lesion area and not larger than 12 disk areas, as confirmed by the CRC. Inclusion was limited to patients who had not received any previously administered, approved or investigational therapy for nAMD in the study eye.

Key exclusion criteria included subretinal hemorrhage in the study eye involving the center of the fovea with size either >50% of the lesion area or >1 disk area; vitreous hemorrhage in the study eye; history of rhegmatogenous retinal detachment in the study eye; subfoveal fibrosis or scarring within the lesion in the study eye, or retinal angiomatous proliferation lesion, or large (>50% of the CNV lesion) subfoveal pigment epithelium detachment as confirmed by the CRC; prior submacular surgery or vitrectomy in the study eye; active infection or inflammation in either eye; and history of chronic therapy with systemic or topical corticosteroids or any intraocular therapy with corticosteroids within the past 6 months. If both eyes met the eligibility criteria, the eye with the worst vision was selected as the study eye.

### Randomization and intervention

Study visits were scheduled at baseline (day 1), day 3, and weeks 1, 4, 8, 12, 16, and 20. At the baseline visit, enrolled patients were randomized using a 3:3:2 ratio to intravitreal administration of abicipar 1 mg, abicipar 2 mg, or ranibizumab 0.5 mg (0.05 mL injection volume for each study drug). The assignment to treatment arms was based on order of enrollment and a computer-generated randomization schedule provided by the sponsor. Abicipar was provided in phosphate-buffered saline at ∼60 mg/mL, stored at −20°C, and diluted at the clinical trial sites to 20 and 40 mg/mL for the 1 mg and 2 mg doses. Ranibizumab 0.5 mg was provided at 10 mg/mL for 0.5-mg doses. A schematic of the study design is shown in Supplementary Fig. S1 (Supplementary Data are available online at www.liebertpub.com/jop).

Patients randomized to the ranibizumab arm received 5 monthly intravitreal injections administered at the baseline visit and at weeks 4, 8, 12, and 16. Patients randomized to abicipar (1 mg or 2 mg) received 3 monthly intravitreal injections administered at the baseline visit and at weeks 4 and 8. Because different storage conditions and dilutions of the study drugs were needed, investigators who performed the injections were unmasked to treatment. However, patients were masked to their treatment assignment, and all efficacy assessments were performed by study personnel who were masked to patients' treatment assignment. To maintain masking, patients in the abicipar treatment arms received a sham injection procedure at weeks 12 and 16.

At week 12 and later visits, patients could be treated with standard of care (SoC) if there was evidence of any active disease, as determined by the investigator. Evidence of active disease included retinal fluid on OCT, or other signs of active CNV including new or persistent subretinal or intraretinal hemorrhage, a decrease in visual acuity from the last visit without another explanation, or leakage or an increase in lesion size relative to the last angiogram on fluorescein angiography. Patients were followed to week 20 or to 4 weeks after escape to SoC, whichever occurred earlier.

### Outcome measures

Efficacy evaluations including BCVA and OCT imaging were performed at all study visits except day 3 (safety visit). BCVA was assessed using a modification of the Early Treatment Diabetic Retinopathy Study (ETDRS) method.^16^ OCT measurements were performed with either a Cirrus (Carl Zeiss Meditec, Jena, Germany) or a Spectralis (Heidelberg Engineering, Heidelberg, Germany) spectral-domain instrument; all measurements for an individual patient were performed with the same instrument. OCT images were graded at the CRC for both CRT (average thickness in the 1-mm diameter central macular subfield) and fluid compartments by readers masked to the treatment assignment. Fluid compartments were defined by the CRC as follows: subretinal fluid (SRF), fluid between the photoreceptor layer and the RPE; cystic intraretinal fluid (CIRF), a cavity within the retina between the photoreceptor layer and the internal limiting membrane; and non-cystic intraretinal fluid (NIRF), diffuse thickening of the retina (“spongiform” fluid) without any visible CIRF or SRF. Fluorescein angiography was performed at screening and week 20.

The primary endpoint was the mean change from baseline in BCVA in the study eye at week 16. Key secondary efficacy measures included mean change in BCVA from baseline, mean change in CRT from baseline, and the proportion of patients with ≥15-letter gain in BCVA from baseline in the study eye at each scheduled follow-up visit. The proportion of patients with stable vision (no loss or a <15-letter loss in BCVA from baseline) in the study eye at each scheduled follow-up visit was also evaluated. In addition, among patients who had at least 1 retinal fluid compartment present at baseline in the study eye on OCT (SRF, CIRF, or NIRF), the proportion who had no fluid compartment present at follow-up (“all dry” patients) was a preplanned secondary endpoint at week 12 and was assessed at each scheduled follow-up visit as an exploratory endpoint.

Safety measures included adverse events (AEs), biomicroscopy and ophthalmoscopy, BCVA, assessment of the study eye postinjection, physical examination, clinical laboratory analysis (hematology, serum chemistry, and urinalysis), and immunogenicity. AE reporting included the seriousness and severity of the event, as well as the action taken and the potential relationship to the study drug. Any serious AE occurring during the study period (beginning with informed consent) and for at least 28 days after the last injection of study drug was to be immediately reported to Allergan and followed up, with the outcome reported. AEs were categorized by preferred terms using the Medical Dictionary for Regulatory Activities (MedDRA) Version 16.1. For biomicroscopy/ophthalmoscopy findings with no severity data collected, any finding that was reported as absent at screening and present during the study was included in the tabulation of findings with a 1+ increase in severity.

### Pharmacokinetic and immunogenicity analyses

Blood samples for pharmacokinetic analyses were collected from patients at selected sites on day 1 (predose and 1 h postdose), day 3, and week 1, and predose at weeks 4, 8, and 12. Serum abicipar concentrations were determined by Charles River Laboratories (Montreal, Canada). In addition, blood samples were collected from all patients on day 1 and weeks 4, 12, and 20, before any administration of study therapy, to assess immunogenicity. The presence of antibodies directed against abicipar and the pegol moiety of abicipar in samples collected from abicipar-treated patients was evaluated by Charles River Laboratories using a validated enzyme-linked immunosorbent assay method. This method involved an initial screening and confirmation of positive samples with competitive binding assays using abicipar or polyethylene glycol (PEG). Samples from patients before abicipar treatment were all negative for anti-abicipar and anti-PEG antibodies. Immunogenicity results were reported as positive or negative and are presented as number and percentage of patients for each treatment arm.

### Statistical analysis

Statistical analysis was performed using SAS version 9.3 software (SAS Institute, Inc., Cary, NC) and a 2-sided alpha level of 0.05. Efficacy measures were evaluated in the modified intent-to-treat (mITT) population of all randomized and treated patients with BCVA or CRT data at baseline and at least 1 postbaseline timepoint. Safety measures were evaluated in the safety population of all treated patients.

Comparisons of baseline characteristics among treatment arms used analysis of variance for age, Chi-square tests or Fisher's exact tests for sex and race, and the Cochran-Mantel-Haenszel method with ridit scores for type of CNV. Study completion rates were compared among treatment arms using the Chi-square test. Baseline BCVA and CRT were compared among treatment arms using analysis of variance with treatment and baseline BCVA strata (<55 letters or ≥55 letters) as factors. The statistical plan for the study called for changes in BCVA and CRT from baseline to be analyzed using analysis of covariance (ANCOVA) models with baseline BCVA or CRT as the covariate. In these preplanned analyses, any efficacy data collected after escape to SoC in the abicipar arms were excluded from analysis and set to missing, and missing values were imputed using the last-observation-carried-forward (LOCF) method. Results of the preplanned analyses of change in BCVA and CRT from baseline are provided in Supplementary Tables S1 and S2.

Subsequently, *post hoc* efficacy analyses were conducted using observed values with no imputation for missing values. The *post hoc* analyses also used more robust mixed model repeated measures (MMRM) analysis of changes in BCVA and CRT from baseline. Data collected from patients in the ranibizumab arm after escape to SoC were included in the analysis, because all of these patients received monthly ranibizumab as the escape therapy. The results of the *post hoc* analyses using observed values only and MMRM analysis of changes in BCVA and CRT from baseline were consistent with the results of the preplanned analyses using LOCF and ANCOVA models of changes in BCVA and CRT from baseline. All efficacy data presented in Results are from *post hoc* analyses, with no imputation for missing values.

Changes in BCVA and CRT from baseline were analyzed using an MMRM model that included treatment group, baseline BCVA or CRT, visit, visit by baseline BCVA or CRT interaction, and treatment by visit interaction as fixed covariates in an unstructured covariance matrix. Statistically significant differences between each abicipar arm and the ranibizumab arm (abicipar arm minus ranibizumab arm) were determined by calculating the 2-sided 95% confidence intervals (CIs) of the differences and *P* values from the MMRM model. The proportion of patients with ≥15-letter BCVA gain, stable vision, and no fluid compartments on OCT was analyzed at each visit using the Fisher's exact test, with a *P* value <0.05 considered statistically significant. REACH stage 3 was not powered to detect statistically significant differences in the primary endpoint. The planned sample size of 64 patients, randomized in a 3:3:2 ratio to abicipar 1 mg, abicipar 2 mg, and ranibizumab 0.5 mg, was determined empirically.

## Results

### Baseline characteristics and patient disposition

A total of 64 treatment-naive patients with nAMD were enrolled at 15 sites in REACH stage 3 and were randomized in a 3:3:2 ratio to study treatment. The mean age of the patients was 76.6 years (range 53–91 years). Overall, 39/64 (61%) patients were female and 62/64 (97%) were white, typical of an nAMD clinical study population. Baseline patient characteristics were similar in the 3 treatment arms (Table 1). There were no statistically significant differences at baseline among the treatment arms in demographics or in BCVA, CRT, or type of CNV in the study eye.

**Table 1.** Baseline Patient and Study Eye Characteristics in the REACH Stage 3 Study

				P *Value*		
*Characteristic*	*Abicipar 1 mg (*n* = 25)*	*Abicipar 2 mg (*n* = 23)*	*Ranibizumab 0.5 mg (*n* = 16)*	*Overall*	*Abicipar 1 mg vs. Ranibizumab*	*Abicipar 2 mg vs. Ranibizumab*
Age, mean (SD), y	76 (10)	78 (6)	77 (9)	0.623		
Gender				0.320		
Female, *n* (%)	18 (72)	13 (57)	8 (50)			
Race				>0.999		
White, *n* (%)	24 (96)	22 (96)	16 (100)			
BCVA, mean (SD), ETDRS letters	58 (13)	59 (14)	60 (16)		0.996	0.488
CRT, mean (SD), μm	526 (165)	466 (126)	463 (95)		0.183	0.967
Type of CNV, *n* (%)					0.507	0.181
Predominantly classic	9 (36)	9 (39)	3 (19)			
Occult (late leakage)	1 (4)	0 (0)	0 (0)			
Occult (fibrovascular PED)	12 (48)	14 (61)	13 (81)			
Occult with serous PED	0 (0)	0 (0)	0 (0)			
Unable to grade	1 (4)	0 (0)	0 (0)			
Other	2 (8)	0 (0)	0 (0)			

PED, pigment epithelium detachment; SD, standard deviation.

Study completion was defined as completion of the week 20 visit or the visit 4 weeks after escape to SoC. Overall, 62/64 (97%) patients completed REACH stage 3. The study completion rate was comparable among treatment arms (*P* = 0.159). Twenty-one (84.0%) patients in the abicipar 1 mg arm, 19 (82.6%) patients in the abicipar 2 mg arm, and 14 (87.5%) patients in the ranibizumab arm completed the study at week 20; whereas 4 (16.0%) patients in the abicipar 1 mg arm, 2 (8.7%) patients in the abicipar 2 mg arm, and 2 (12.5%) patients in the ranibizumab arm completed the study at week 16, at 4 weeks after escape to SoC. Two (8.7%) patients in the abicipar 2 mg arm discontinued REACH stage 3, one due to an AE. The other patient withdrew consent because blood draws were very uncomfortable for the patient. All randomized patients received the assigned study treatment and were included in the mITT, per-protocol, and safety populations for analysis.

### Escape treatment

During REACH stage 3, 14/25 (56.0%) patients treated with abicipar 1 mg, 11/23 (47.8%) patients treated with abicipar 2 mg, and 10/16 (62.5%) treated with ranibizumab demonstrated active disease at or after week 12 and were prescribed escape treatment. For patients in the abicipar arms, efficacy data collected after escape treatment were excluded from analysis; the number of patients in the abicipar 1 mg and 2 mg arms included in the efficacy analyses (i.e., had not escaped to SoC and did not have missing data) was 24 (96%) and 22 (96%) at week 12, 19 (76%) and 19 (83%) at week 16, and 14 (56%) and 13 (57%) at week 20, respectively.

Because the randomization schedule was uneven for ranibizumab and all patients who met the criteria for escape in the ranibizumab arm were prescribed monthly ranibizumab as the escape therapy, efficacy data collected for patients in the ranibizumab arm after escape were included in the primary analyses; the number of patients in the ranibizumab arm included in the primary analyses presented was 16 (100%) at week 12, 16 (100%) at week 16, and 14 (88%) at week 20. However, sensitivity analysis was conducted that excluded all efficacy data from patients in all treatment arms after escape to SoC (i.e., data collected after patients in the ranibizumab arm escaped to SoC ranibizumab treatment were also excluded from analysis). In the sensitivity analysis, the number of patients in the ranibizumab arm was 16 (100%) at week 12, 10 (62.5%) at week 16, and 7 (43.8%) at week 20. Supplementary Fig. S2 shows the results of the sensitivity analysis for each efficacy endpoint. Results of the sensitivity analysis were confirmatory, and similar conclusions were drawn from the primary and sensitivity analyses.

### Efficacy outcomes

**FIG. 1.**
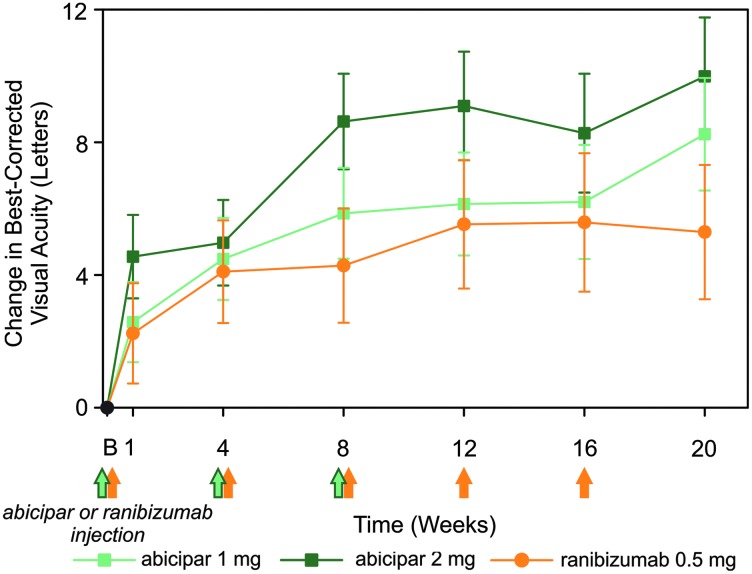
Change in BCVA (letters) from baseline in the modified intent-to-treat population of patients treated with abicipar 1 mg (*light green lines* and *squares*; *n* = 25), abicipar 2 mg (*dark green lines* and *squares*; *n* = 23), or ranibizumab 0.5 mg (*orange lines* and *circles*; *n* = 16). Data shown are least-squares means ± standard errors from the mixed-effects model for repeated measures. There were no statistically significant differences between abicipar 1 mg or 2 mg and ranibizumab 0.5 mg in change in BCVA from baseline. *Green* and *orange arrows* indicate when the 3 abicipar injections or 5 ranibizumab injections were administered. Mean ± standard error of the mean BCVA at baseline for the abicipar 1 mg, abicipar 2 mg, and ranibizumab 0.5 mg arms was 58.4 ± 3.7, 58.5 ± 3.8, and 60.4 ± 4.1 letters, respectively. B, baseline. BCVA, best-corrected visual acuity.

Improvements in BCVA were observed in all treatment arms with no significant differences between the abicipar and ranibizumab arms (Fig. 1). At week 16 (8 weeks after the last abicipar and 4 weeks after the last ranibizumab intravitreal administration), the least-squares (LS) mean change in BCVA from baseline was +6.2, +8.3, and +5.6 letters in the abicipar 1 mg, abicipar 2 mg, and ranibizumab 0.5 mg arms, respectively. At week 20 (12 weeks after the last injection of abicipar and 4 weeks after the last intravitreal injection of ranibizumab), the LS mean change in BCVA from baseline was +8.2, +10.0, and +5.3 letters in the abicipar 1 mg, abicipar 2 mg, and ranibizumab 0.5 mg arms, respectively (Fig. 1). Summary statistics for the observed data are provided in Supplementary Table S3.

**FIG. 2.**
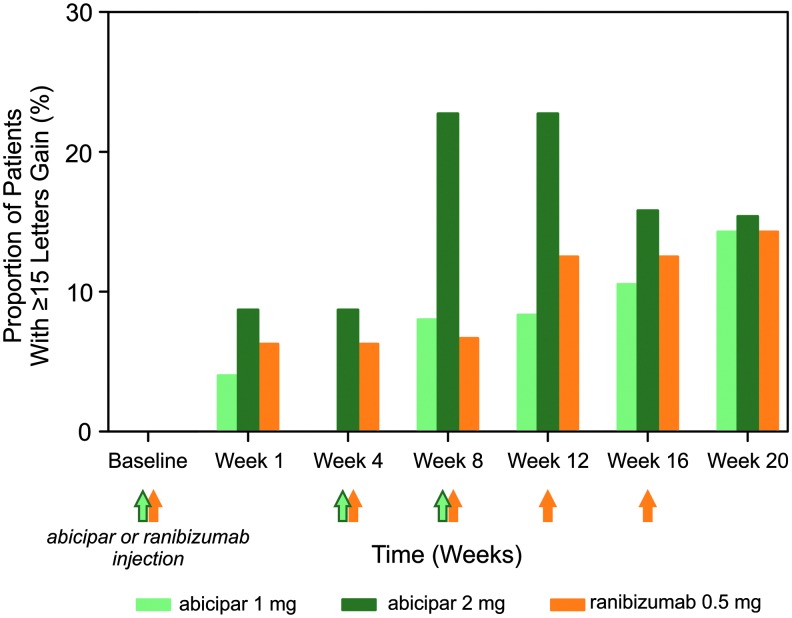
Proportion (%) of patients in the modified intent-to-treat population gaining ≥15 letters of best-corrected visual acuity from baseline after treatment with abicipar 1 mg (*light green bars*; *n* = 25), abicipar 2 mg (*dark green bars*; *n* = 23), or ranibizumab 0.5 mg (*orange bars*; *n* = 16). Proportions are calculated for patients with data available who had not been rescued (unless rescued with ranibizumab in the ranibizumab group). There were no statistically significant differences between treatment arms. *Green* and *orange arrows* indicate when the 3 abicipar injections or 5 ranibizumab injections were administered.

Achievement of 3-line gains in vision and achievement of stable vision were also comparable between the abicipar and ranibizumab arms. The proportion of patients gaining 15 letters of vision in BCVA among those who remained in REACH stage 3 and had not been rescued (unless rescued with ranibizumab in the ranibizumab group) was 10.5%, 15.8%, and 12.5% in the abicipar 1 mg, abicipar 2 mg, and ranibizumab 0.5 mg arms, respectively, at week 16 (Fig. 2). These BCVA gains at week 16 were achieved 8 weeks after the last abicipar injection and 4 weeks after the last ranibizumab injection. At week 20, 12 weeks after the last abicipar 1 mg and 2 mg injections and 4 weeks after the last ranibizumab injection, the proportion of patients achieving ≥15-letter gain was 14.3%, 15.4%, and 14.3%, respectively (Fig. 2). The proportion of patients with stable vision, defined as no loss or a <15-letter loss in BCVA from baseline, was also evaluated, as this was the primary endpoint in the registration studies for ranibizumab and aflibercept in nAMD.^4,5,17^ At week 16, the proportion of patients with stable vision among those who remained in REACH stage 3 and had not been rescued (unless rescued with ranibizumab in the ranibizumab group) was 100% in the abicipar 1 mg and 2 mg arms and 93.8% in the ranibizumab 0.5 mg arm. At week 20, the proportion was 100% in all 3 treatment arms.

**FIG. 3.**
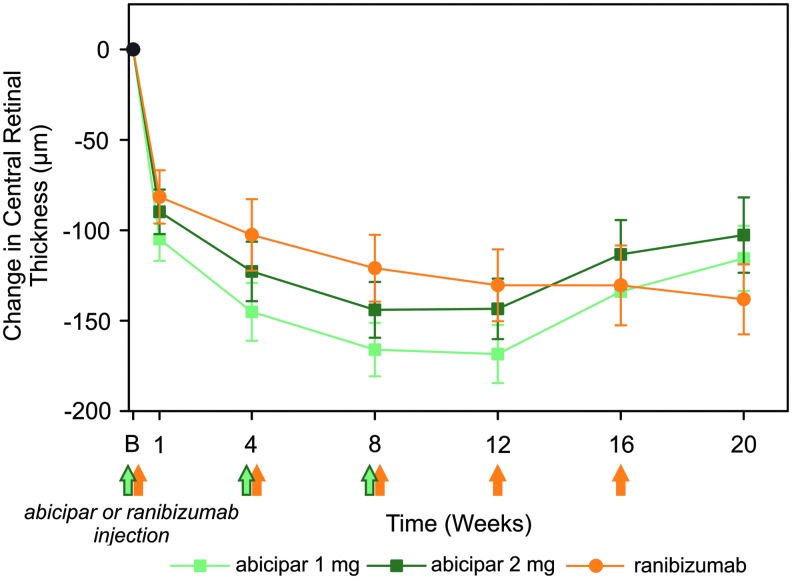
Change from baseline in CRT (μm) in the modified intent-to-treat population of patients treated with abicipar 1 mg (*light green lines* and *squares*; *n* = 25), abicipar 2 mg (*dark green lines* and *squares*; *n* = 23), or ranibizumab 0.5 mg (*orange lines* and *circles*; *n* = 16). Data shown are least-squares means ± standard errors from the mixed-effects model for repeated measures. There were no statistically significant differences between abicipar 1 mg or 2 mg and ranibizumab 0.5 mg in change in CRT from baseline. *Green* and *orange arrows* indicate when the 3 abicipar injections or 5 ranibizumab injections were administered. Mean ± standard error of the mean CRT at baseline for the abicipar 1 mg, abicipar 2 mg, and ranibizumab 0.5 mg arms was 526 ± 33, 466 ± 26, and 463 ± 24 μm, respectively. B, baseline. CRT, central retinal thickness.

In REACH stage 3, abicipar 1 mg and 2 mg were similarly effective compared with ranibizumab in reducing the anatomical endpoint of CRT. The LS mean CRT reduction from baseline was 134, 113, and 131 μm at week 16 and 116, 103, and 138 μm at week 20 in the abicipar 1 mg, abicipar 2 mg, and ranibizumab 0.5 mg arms, respectively (Fig. 3). Summary statistics for the observed data are provided in Supplementary Table S3. The proportion of patients who had complete resolution of the retinal fluid compartments of SRF, CIRF, and NIRF on OCT during REACH stage 3 was also evaluated. An “all dry” retina status was defined as complete resolution of all 3 compartments of fluid (any compartment with missing data was considered to be dry). SD-OCT images showing the resolution of retinal fluid compartments in a patient in the abicipar 2 mg arm are presented in Supplementary Fig. S3.

**FIG. 4.**
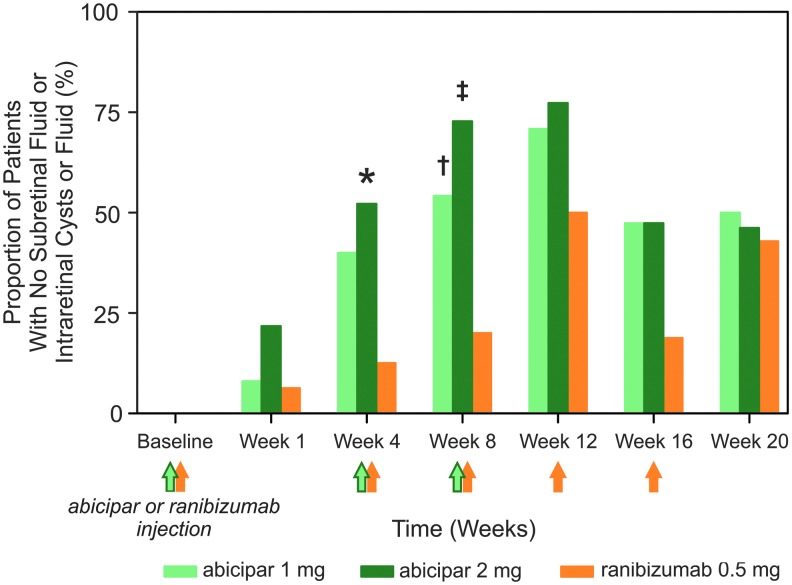
Proportion (%) of patients in the modified intent-to-treat population achieving resolution of the 3 fluid compartments of subretinal fluid, intraretinal cyst, and intraretinal fluid (an “all dry” retina) after treatment with abicipar 1 mg (*light green bars*; *n* = 25), abicipar 2 mg (*dark green bars*; *n* = 23), or ranibizumab 0.5 mg (*orange bars*; *n* = 16). Proportions are calculated for patients with data available who had not been rescued (unless rescued with ranibizumab in the ranibizumab group). *Green* and *orange arrows* indicate when the 3 abicipar or 5 ranibizumab injections were administered. ^*^P = 0.017 versus ranibizumab; ^**†**^*P* = 0.049 versus ranibizumab, ^**‡**^*P* = 0.003 versus ranibizumab.

All patients in each treatment arm had at least 1 form of retinal fluid present at baseline. At weeks 4 and 8, abicipar 1 mg and 2 mg resolved fluid accumulation more effectively than ranibizumab 0.5 mg (Fig. 4). At week 12, the proportion of patients with an “all dry” status among those who remained in REACH stage 3 and had not been rescued (unless rescued with ranibizumab in the ranibizumab group) was 70.8% and 77.3% in the abicipar 1 mg and 2 mg arms compared with 50.0% in the ranibizumab 0.5 mg arm. At week 16 (8 weeks after the last abicipar injection), the proportion of patients with complete resolution of these fluid compartments was 47.4% and 47.4% in the abicipar 1 mg and 2 mg arms compared with 18.8% in the ranibizumab arm (4 weeks after intravitreal ranibizumab). At the end of REACH stage 3 (week 20, 12 weeks after the last abicipar injection and 4 weeks after the last intravitreal ranibizumab injection), the proportion of patients with complete resolution of fluid was 50.0%, 46.2%, and 42.9% in the abicipar 1 mg, abicipar 2 mg, and ranibizumab 0.5 mg arms, respectively. Similar results were obtained in a supplementary analysis that used all observed data in all treatment arms and included data from patients after escape to SoC (Supplementary Fig. S4).

Fluorescein angiography showed decreases in the mean area of CNV at week 20, with no significant differences between the abicipar groups and the ranibizumab group.

### Pharmacokinetics and immunogenicity

To evaluate the potential for abicipar to inhibit systemic VEGF action and elicit production of anti-abicipar/pegol antibodies, pharmacokinetic and immunogenicity testing was conducted. The concentration of free abicipar was measurable in serum samples collected on day 3 in both abicipar treatment arms (mean ± standard deviation concentration of 0.55 ± 0.35 nM in the abicipar 1 mg arm and 1.05 ± 0.60 nM in the abicipar 2 mg arm), as well as in serum samples collected at week 1 in the abicipar 2 mg arm (mean concentration of 0.43 ± 0.33 nM). At all other timepoints, serum concentrations of free abicipar were below the limit of quantitation (0.3 nM) in all samples. Pharmacokinetic parameters such as t_1/2_ could not be calculated because of the limited number of timepoints at which concentrations of abicipar could be determined.

Anti-abicipar antibodies were detectable in blood samples from 14/25 (56.0%) patients in the abicipar 1 mg arm and in 3/23 (13.0%) patients in the abicipar 2 mg arm. Antibodies directed to the pegol portion of the protein (anti-PEG antibodies) were detectable in 2/25 (8.0%) patients in the abicipar 1 mg arm after the first injection of abicipar 1 mg. No patients in the abicipar 2 mg arm had anti-PEG antibodies at any timepoint, and all patients in the abicipar 1 mg arm were negative for anti-PEG antibodies at their last visit with immunogenicity data, indicating a transient antibody response against the pegol moiety of abicipar. A relationship between the presence of anti-abicipar antibodies and safety and/or efficacy data could not be established.

### Safety outcomes

The overall incidence of AEs in REACH stage 3 was 15/25 in the abicipar 1 mg arm, 10/23 in the abicipar 2 mg arm, and 9/16 in the ranibizumab 0.5 mg arm. Most events were mild or moderate in severity. Treatment-related AEs were reported in 10/25, 4/23, and 3/16 of patients in the abicipar 1 mg, abicipar 2 mg, and ranibizumab 0.5 mg arms, respectively. With the exception of an AE reported as iron deficiency in the abicipar 2 mg arm, all of these AEs were ocular. The most common ocular AEs (reported in ≥2 patients in any treatment arm) were vitreous floaters, vitreous detachment, retinal hemorrhage, eye pain, conjunctival hemorrhage, and macular scar (Table 2). Both events of macular scar in the ranibizumab arm and 4 events of retinal hemorrhage (2 in the abicipar 1 mg arm and 2 in the ranibizumab arm) were reported to be unrelated to the study drug or injection. An additional event of retinal hemorrhage in the abicipar 1 mg arm was reported to be related to the injection. No deaths or other serious AEs were reported in any treatment arm.

**Table 2.** Adverse Events in the Study Eye in the REACH Stage 3 Study

*AE*^a^	*Number (%) of patients*		
	*Abicipar 1 mg (*n* = 25)*	*Abicipar 2 mg (*n* = 23)*	*Ranibizumab 0.5 mg (*n* = 16)*
Overall incidence^b^	11 (44.0)	7 (30.4)	5 (31.3)
Vitreous floaters	3 (12.0)	1 (4.3)	1 (6.3)
Vitreous detachment	2 (8.0)	2 (8.7)	0
Retinal hemorrhage	3 (12.0)	0	2 (12.5)
Eye pain	1 (4.0)	2 (8.7)	1 (6.3)
Conjunctival hemorrhage	2 (8.0)	0	0
Macular scar	0	0	2 (12.5)

^a^ All AEs reported in the study eye for 2 or more patients in any treatment arm are listed.

^b^ Patients with 1 or more AEs in the study eye.

AE, adverse event.

Intraocular inflammation (IOI) AEs were reported in 5 patients (3 [12.0%] in the abicipar 1 mg arm, 2 [8.7%] in the abicipar 2 mg arm, and none in the ranibizumab arm). In the abicipar 1 mg arm, these AEs included iritis (reported as iritis; moderate; after the third injection), uveitis (reported as panuveitis; moderate; after the second injection), and vitritis (reported as vitritis; severe; after the third injection). In the abicipar 2 mg arm, these AEs included iritis (reported as iritis; mild; after the third injection) and choroiditis (reported as posterior uveitis; moderate; after the second injection). The patient with vitritis did not receive any treatment and the AE resolved; the other 4 patients with IOI received topical corticosteroid treatment, and 2 of these patients also received 1 week of oral corticosteroid treatment. The patient in the abicipar 2 mg arm with choroiditis discontinued from the study because of the AE; the choroiditis resolved without sequelae after treatment with a topical corticosteroid. All AEs of IOI resolved without sequelae within 26–84 days, and none was associated with a sustained loss in vision (Table 3).

**Table 3.** Visual Outcomes After Intraocular Inflammation Adverse Events

*Intraocular inflammation AE (MedDRA preferred term)*	*Treatment arm*	*Worst change from baseline BCVA after AE (letters)*	*BCVA change from baseline at escape to SoC or study exit (letters)*
Iritis	Abicipar 1 mg	−5	−1
Uveitis	Abicipar 1 mg	−2	+4
Vitritis	Abicipar 1 mg	+14	+22
Iritis	Abicipar 2 mg	NA	+2
Choroiditis	Abicipar 2 mg	−19	+7

AE, adverse event; BCVA, best-corrected visual acuity; MedDRA, medical dictionary for regulatory activities; NA, not available; SoC, standard of care.

No Antiplatelet Trialists Collaboration arterial thromboembolic events (APTC ATEs; including nonfatal myocardial infarction, nonfatal stroke, vascular death, and death of unknown cause)^18^ were reported in REACH stage 3. The only systemic AE potentially related to systemic VEGF inhibition reported in REACH stage 3 was hypertension. An AE of hypertension was reported in 1 patient in the abicipar 2 mg arm and 1 patient in the ranibizumab 0.5 mg arm, and both were deemed not related to study treatment by the investigator. No hypertension AEs were reported in the abicipar 1 mg arm.

Overall, 19/25 (76.0%) patients in the abicipar 1.0 mg arm, 15/23 (65.2%) patients in the abicipar 2.0 mg arm, and 10/16 (62.5%) patients in the ranibizumab arm had a ≥1-grade increase from baseline in the severity of any biomicroscopy or ophthalmoscopy finding during the study. A ≥1-grade increase in the severity of detachment of macular RPE (no findings at screening and positive findings during the study) was reported in 2 (8.0%) patients in the abicipar 1 mg arm and no patients in either the abicipar 2 mg arm or the ranibizumab arm.

## Discussion

Sudden vision loss in advanced AMD is attributed in large part to nAMD, and anti-VEGF agents such as ranibizumab have changed clinical practice for the treatment of this disease.^1^ In registration studies of anti-VEGF treatment in nAMD,^4,5,17^ vision was improved or stabilized in more than 90% of patients, and the effects on vision were associated with significantly improved vision-related quality of life.^19,20^ Accordingly, intravitreal injections of anti-VEGF drugs have become the mainstay of current treatment for nAMD.^1^ A drawback to this treatment, however, is the need for frequent intravitreal injections. In clinical trials, monthly injections of anti-VEGF therapies have provided the most robust efficacy outcomes.^21^ Therefore, there is currently an unmet medical need for a therapy that can maintain maximal efficacy while requiring less frequent injections. Not only would such a therapy remove a significant treatment burden on patients and their healthcare providers, but it would also decrease the risk of complications associated with intravitreal injections such as endophthalmitis, retinal detachment, and elevated intraocular pressure. The data from REACH stage 3 suggest that, in comparison to ranibizumab, abicipar elicits similar visual acuity gains with a reduced number of injections (3 vs. 5 in this short-duration study). The data also demonstrate that for many patients, effects on BCVA are maintained 12 weeks after the last injection of abicipar, suggesting greater duration of effect, and indicating that abicipar could provide similar efficacy compared with ranibizumab, but with reduced burden of visits for patients and healthcare providers.

In REACH stage 3, abicipar induced rapid increases in BCVA from baseline, achieving the maximal effect by week 8, after 2 intravitreal injections, and demonstrating sustained efficacy at week 16, the timepoint for the primary endpoint. It is important to note that week 16 corresponded to 8 weeks after the last abicipar injection. At week 20, mean change from baseline BCVA was +8.2 and +10.0 letters with abicipar 1 mg and 2 mg, respectively, compared with +5.3 letters for ranibizumab. These data suggest that an 8-week or 12-week dosing interval for abicipar may be feasible to sustain efficacy outcomes.

Differences in the selection criteria for patient populations of this study and the ranibizumab registration studies (e.g., allowed lesion size, and inclusion or exclusion of patients with RAP, large pigment epithelial detachment, and previous treatment) can make it difficult to compare results between studies. In the ranibizumab registration studies, the distribution of lesion types in the ranibizumab 0.5 mg treatment arm was 96% predominantly classic, 4% minimally classic, and 0% occult in the ANCHOR study^4^ and 0% predominantly classic, 38% minimally classic, and 62% occult in the MARINA study.^5^ In the ranibizumab 0.5 mg treatment arm in REACH stage 3, the proportion of patients with predominantly classic lesions was 19% and the proportion of patients with occult lesions was 81%; due to the similarities in the distribution of lesion type, the MARINA study may be the more relevant comparator study for the ranibizumab outcomes in REACH stage 3. Mean BCVA change from baseline with monthly injections of ranibizumab was +5.6 and +5.3 letters at weeks 16 and 20 in REACH stage 3, similar to the +6.1 and +6.2 letters seen in MARINA for ranibizumab.

Evaluation of retinal anatomy using OCT suggested that abicipar effects were maintained through 12 weeks after the final abicipar administration, with CRT values for abicipar 1 and 2 mg that were comparable to those obtained with ranibizumab administered monthly. However, change in CRT is a relatively insensitive measure of the effect of anti-VEGF treatment on exudation, and it is not well correlated with recovery of vision in nAMD.^22^ Anatomical abnormalities in addition to CNV lesions and exudation, such as photoreceptor and RPE cell dropout and fibrosis, also contribute to abnormal retinal thickness. These would not resolve with anti-VEGF treatment.

SD-OCT visualization of the presence or absence of specific fluid compartments that form in patients with nAMD, including SRF, CIRF, NIRF, and pigment epithelial detachments, avoids the limitations of CRT measurements.^23,24^ Therapeutic effects of treatment may be reflected by changes in these fluid compartments, and there is ongoing discussion of the importance of these anatomical changes.^23,25,26^ The presence of CIRF has been significantly associated with poor visual recovery.^27,28^ Not all patients have all 3 of these fluid compartments, but the presence of SRF, NIRF, and CIRF is commonly used in clinical retina practice to guide anti-VEGF treatment. Therefore, the complete resolution of these 3 retinal fluid compartments may be a relatively sensitive assessment for comparing different therapies and dosing frequencies. This was evaluated as an exploratory endpoint in REACH stage 3, where resolution of all 3 fluid compartments appeared to correlate with improvements in BCVA. The proportion of patients with an “all dry” status was significantly higher in the abicipar 2 mg arm than in the ranibizumab arm at week 4 and significantly higher in both abicipar arms than in the ranibizumab arm at week 8, suggesting that abicipar may be more effective than ranibizumab in achieving an “all dry” retina status during the early phase of treatment. Throughout the remainder of REACH stage 3, the proportion of patients with an “all dry” status was similar in the abicipar arms compared with the ranibizumab arm, despite the greater frequency of injections administered in the ranibizumab arm.

With respect to safety, abicipar was well tolerated, as the overall incidence of AEs in REACH stage 3 for abicipar (1 and 2 mg) was comparable to that for ranibizumab 0.5 mg, and most events were mild or moderate in severity. No serious AEs of IOI were reported, and only 1 AE of IOI resulted in discontinuation from REACH stage 3. In all cases, AEs of IOI resolved without sequelae. The etiology of IOI in abicipar-treated patients and the manufacturing process used for abicipar will continue to be investigated. Overall, the rate of IOI with abicipar in REACH stage 3 was similar to that reported with ranibizumab in early clinical trials for the treatment of nAMD.^4,5^

In addition to ocular AEs, systemic AEs were also monitored, particularly those potentially related to systemic VEGF inhibition; these included hypertension. One AE of hypertension (1/23; 4.3%) was reported in the abicipar 2 mg arm. Evaluation of systemic levels of abicipar suggested no association with the reported hypertensive event. One AE of hypertension was also reported in the ranibizumab arm (1/16; 6.3%). Of note, systemic concentrations of ranibizumab have been demonstrated to be low, with little effect on plasma VEGF concentrations.^29^

Weaknesses of REACH stage 3 include the limited number of enrolled patients and the relatively short duration of the study. Because the study protocol had broad criteria for active disease at or after week 12, a substantial number of patients in each treatment arm were prescribed escape therapy. Strengths of this study include the exploration of the durability of abicipar effects and the evaluation of complete resolution of retinal fluid compartments, in addition to CRT and BCVA, as efficacy measures.

In conclusion, abicipar provided marked improvements in BCVA and CRT in REACH stage 3. A dosing regimen of 3 initial monthly injections provided long-lasting, beneficial treatment effects in most patients for 8–12 weeks after the last (third) abicipar injection. These effects were similar to those of a fixed monthly ranibizumab 0.5-mg regimen. In other words, similar BCVA and CRT improvement was achieved with a total of 3 abicipar injections *versus* 5 ranibizumab injections in REACH stage 3. Abicipar 1 mg and 2 mg performed as well or better than ranibizumab 0.5 mg in providing patients with an “all dry” retina (no NIRF, CIRF, or SRF), and as with the primary and secondary outcomes, it did so with a reduced number of injections. Overall, abicipar was well tolerated; the incidence of treatment-related AEs was comparable to ranibizumab. No serious AEs were reported. IOI was observed in the abicipar-treated arms at rates similar to those reported in the registration studies for ranibizumab. IOI AEs responded well to treatment and resolved without sequelae; and in 1 patient, the IOI AE resolved without any treatment (Table 3). With respect to systemic AEs, no APTC ATEs were observed.

Overall, the REACH stage 3 data suggest that abicipar could achieve similar efficacy to monthly injections of ranibizumab 0.5 mg, but with a reduced number of injections and dosing intervals of 8 or 12 weeks, consistent with greater durability of action. These data represent the foundation for larger and longer-duration studies with abicipar to evaluate its potential as an effective therapy for nAMD. Global phase 3 clinical trials of abicipar in patients with nAMD are ongoing (NCT02462486, NCT02462928).

## Supplementary Material

Supplemental data

Supplemental data

Supplemental data

Supplemental data

Supplemental data

Supplemental data

Supplemental data

## The REACH Study Group Principal Investigators

Jennifer Arnold (Paramatta, New South Wales, Australia), Robert Avery (Santa Barbara, CA), Michael Bennett (Honolulu, HI), Brian Berger (Austin, TX), Barbara Blodi (Madison, WI), David Boyer* (Beverly Hills, CA), David Brown (Houston, TX), David Callanan* (Arlington, TX), Karl Csaky* (Dallas, TX), Francois Devin (Marseille, France), David W. Faber* (Salt Lake City, UT), Samantha Fraser-Bell* (Sydney, New South Wales, Australia), Joseph R. Ferencz (Kfar-Saba, Israel), Carsten Framme (Bern, Switzerland), David Gaucher (Strasbourg, France), Mark Gillies (Sydney, New South Wales, Australia), Michaella Goldstein (Tel Aviv, Israel), Robyn Guymer* (East Melbourne, Australia), Jeffrey Heier (Boston, MA), Frank Holz (Bonn, Germany), Rahul N. Khurana* (Mountain View, CA), Gregg Kokame* (Honolulu, HI), Jean-Francois Korobelnik (Bordeaux, France), Derek Kunimoto* (Phoenix, AZ), Paolo Lanzetta (Udine, Italy), Chris Patrick Lohmann (Munich, Germany), Raj Maturi* (Indianapolis, IN), Saddek Mohand-Said (Paris, France), George Papastergiou (Honolulu, HI), Sunil Patel* (Abilene, TX), Daniel Pauleikhoff (Munster, Germany), Ayala Pollack* (Rehovot, Israel), Christian Pruente (Binningen, Switzerland), Steven Rose* (Rochester, NY), Irit Rosenblatt (Petach Tikva, Israel), Ursula Schmidt-Erfurth (Vienna, Austria), Ashish Sharma* (Fort Myers, FL), Lawrence Singerman (Cleveland, OH), Eric Souied (Creteil, France), Giovanni Staurenghi (Milan, Italy), Paul Tornambe* (Poway, CA), Charles Wykoff (The Woodlands, TX), and Martin Zinkernagel (Bern, Switzerland).
