# Supplementary material for: Double-Masked, Randomized, Phase 2 Evaluation of Abicipar Pegol (an Anti-VEGF DARPin Therapeutic) in Neovascular Age-Related Macular Degeneration
Source: J Ocul Pharmacol Ther. 2018 Dec 6;34(10):700–9. doi: 10.1089/jop.2018.0062 (PMC6306670; doi:10.1089/jop.2018.0062)
Supplement: Supplemental data [file Supp_Fig1.pdf]

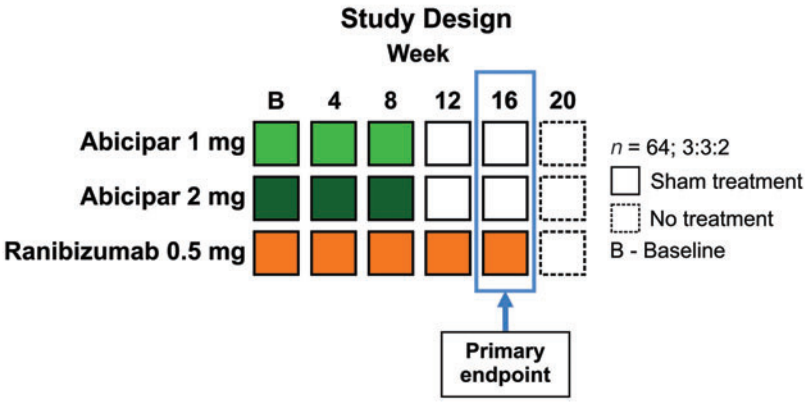

SUPPLEMENTARY FIG. S1. REACH stage 3 study design. A total of 64 patients were randomized in a 3:3:2 ratio to the 3 treatment arms: abicipar 1 mg (*light green*; $n = 25$), abicipar 2 mg (*dark green*; $n = 23$), and ranibizumab 0.5 mg (*orange*; $n = 16$). Abicipar injections were given at baseline and weeks 4 and 8. In contrast, ranibizumab injections were administered at baseline and then every 4 weeks, for a total of 3 abicipar injections and 5 ranibizumab injections. To maintain masking, sham treatments were provided at weeks 12 and 16 to the patients allocated to the abicipar arms (*white boxes*). No treatment (*boxes* with *dashed line borders*) was administered at week 20. The primary endpoint of change from baseline best-corrected visual acuity was at week 16, 8 weeks after the last abicipar injection and 4 weeks after the week 12 ranibizumab injection. Final evaluations were at week 20 (study exit), 12 weeks after the last abicipar injection and 4 weeks after the last ranibizumab injection. B, baseline.
