# Supplementary material for: Double-Masked, Randomized, Phase 2 Evaluation of Abicipar Pegol (an Anti-VEGF DARPin Therapeutic) in Neovascular Age-Related Macular Degeneration
Source: J Ocul Pharmacol Ther. 2018 Dec 6;34(10):700–9. doi: 10.1089/jop.2018.0062 (PMC6306670; doi:10.1089/jop.2018.0062)
Supplement: Supplemental data [file Supp_Table1.pdf]

Supplementary Data

Supplementary Table S1. Mean (SD) Change in Best-Corrected Visual Acuity from Baseline, Early Treatment Diabetic Retinopathy Study Letters

Visit	*Abicipar 1 mg* (n = *25*)	*Abicipar 2 mg* (n = *23*)	*Ranibizumab 0.5 mg* (n = *16*)	P *value for abicipar 1 mg vs. ranibizumab*	P *value for abicipar 2 mg vs. ranibizumab*
Week 1	2.6 (6.5)	4.6 (6.3)	2.1 (5.7)	0.871	0.238
Week 4	4.6 (6.0)	5.0 (7.4)	3.9 (6.0)	0.859	0.644
Week 8	5.9 (6.4)	8.4 (7.5)	3.9 (7.7)	0.444	0.068
Week 12	6.2 (6.9)	8.9 (7.8)	5.3 (10.1)	0.835	0.203
Week 16[a]	6.3 (7.8)	8.2 (7.9)	5.3 (11.1)	0.831	0.348
Week 20	7.1 (7.9)	9.0 (8.0)	4.7 (10.5)	0.462	0.144

Missing values were imputed by using the last-observation-carried-forward (LOCF) method. Data after administration of standard-of-care rescue treatment in the abicipar arms were set to missing and imputed by using LOCF. Data after administration of rescue treatment in the ranibizumab arm were included in the analysis, because all rescued patients in the ranibizumab arm received ranibizumab as the standard-of-care rescue treatment. Mean BCVA values were compared between each abicipar arm and the ranibizumab arm using a 2-way analysis of covariance model with treatment and baseline BCVA strata (<55 letters or ≥55 letters) as factors and baseline BCVA as the covariate. There were no statistically significant differences between abicipar 1 mg or 2 mg and ranibizumab 0.5 mg in mean change in BCVA from baseline. Baseline mean (SD) BCVA was 58.4 (13.5) letters, 58.5 (14.3) letters, and 60.4 (16.4) letters in the abicipar 1 mg, abicipar 2 mg, and ranibizumab 0.5 mg arms, respectively.

[a]The primary endpoint was mean change in BCVA from baseline at week 16.

BCVA, best-corrected visual acuity; ETDRS, Early Treatment Diabetic Retinopathy Study; SD, standard deviation.
