# Supplementary material for: Double-Masked, Randomized, Phase 2 Evaluation of Abicipar Pegol (an Anti-VEGF DARPin Therapeutic) in Neovascular Age-Related Macular Degeneration
Source: J Ocul Pharmacol Ther. 2018 Dec 6;34(10):700–9. doi: 10.1089/jop.2018.0062 (PMC6306670; doi:10.1089/jop.2018.0062)
Supplement: Supplemental data [file Supp_Table2.pdf]

Supplementary Table S2. Mean (SD) Change in Central Retinal Thickness from Baseline, μm

Visit	*Abicipar 1 mg* (n=*25*)	*Abicipar 2 mg* (n=*23*)	*Ranibizumab 0.5 mg* (n=*16*)	P *value for abicipar 1 mg vs. ranibizumab*	P *value for abicipar 2 mg vs. ranibizumab*
Week 1	−122.9 (97.0)	−87.5 (61.9)	−78.4 (55.9)	0.264	0.677
Week 4	−168.3 (137.1)	−119.8 (68.5)	−98.4 (65.2)	0.120	0.440
Week 8	−193.7 (129.9)	−140.3 (77.0)	−115.4 (76.7)	0.059	0.324
Week 12	−194.8 (129.3)	−139.6 (88.4)	−125.8 (89.8)	0.168	0.629
Week 16	−161.0 (136.4)	−110.6 (91.9)	−126.7 (90.0)	0.954	0.538
Week 20	−147.0 (133.4)	−101.5 (92.8)	−124.5 (95.4)	0.743	0.395

Missing values were imputed by using the last-observation-carried-forward (LOCF) method. Data after administration of standard-of-care rescue treatment in the abicipar arms were set to missing and imputed by using LOCF. Data after administration of rescue treatment in the ranibizumab arm were included in the analysis, because all rescued patients in the ranibizumab arm received ranibizumab as the standard-of-care rescue treatment. Mean CRT values were compared between each abicipar arm and the ranibizumab arm using a 2-way analysis of covariance model with treatment and baseline best-corrected visual acuity strata (<55 letters or ≥55 letters) as factors and baseline CRT as the covariate. There were no statistically significant differences between abicipar 1 mg or 2 mg and ranibizumab 0.5 mg in mean change in CRT from baseline. Baseline mean (SD) CRT was 526.1 (165.1) μm, 466.0 (126.0) μm, and 463.3 (94.6) μm in the abicipar 1 mg, abicipar 2 mg, and ranibizumab 0.5 mg arms, respectively.

CRT, central retinal thickness; SD, standard deviation.
