# Supplementary material for: Double-Masked, Randomized, Phase 2 Evaluation of Abicipar Pegol (an Anti-VEGF DARPin Therapeutic) in Neovascular Age-Related Macular Degeneration
Source: J Ocul Pharmacol Ther. 2018 Dec 6;34(10):700–9. doi: 10.1089/jop.2018.0062 (PMC6306670; doi:10.1089/jop.2018.0062)
Supplement: Supplemental data [file Supp_Fig2.pdf]

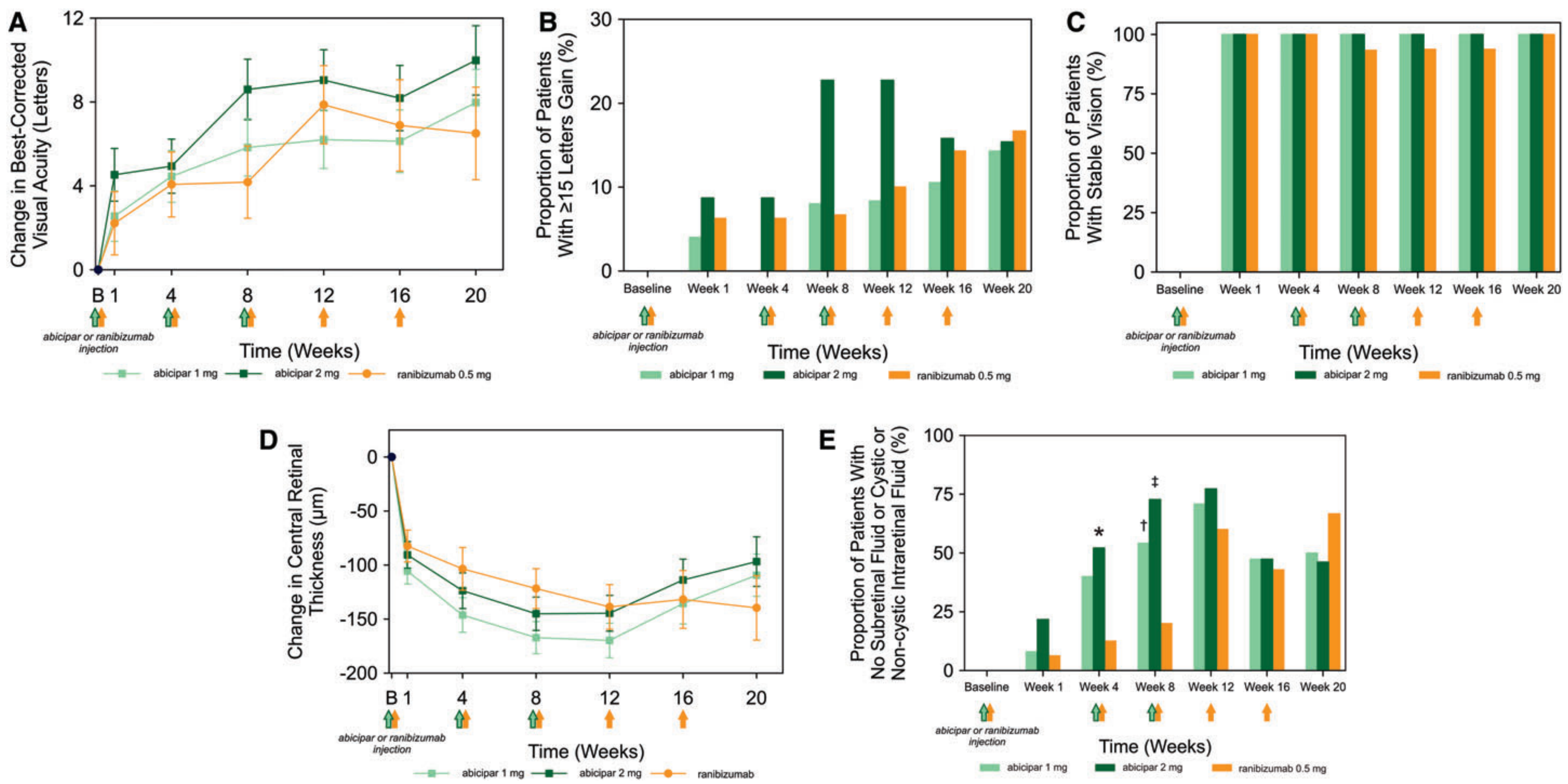

SUPPLEMENTARY FIG. S2. Sensitivity analyses excluding all data from patients in all treatment arms after escape to standard of care. (**A**) Change in BCVA from baseline. Data shown are least-squares means ± standard errors from the mixed-effects model for repeated measures. (**B**) Proportion of patients with ≥15-letter improvement in BCVA from baseline. Proportions are calculated for patients with data available who had not been rescued. (**C**) Proportion of patients with stable vision. Proportions are calculated for patients with data available who had not been rescued. (**D**) Change in CRT from baseline. Data shown are least-squares means ± standard errors from the mixed-effects model for repeated measures. (**E**) Proportion of patients with an ‘‘all dry’’ retinal fluid status. Proportions are calculated for patients with data available who had not been rescued. *Green* and *orange arrows* indicate when the 3 abicipar injections or 5 ranibizumab injections were administered. There were no statistically significant differences between abicipar 1 mg or 2 mg and ranibizumab 0.5 mg in change in BCVA from baseline, the proportion of patients with ≥15-letter BCVA gains, the proportion of patients with stable vision, or change in CRT from baseline. *$P = 0.017$ versus ranibizumab, †$P = 0.049$ versus ranibizumab, ‡$P = 0.003$ versus ranibizumab. BCVA, best-corrected visual acuity; CRT, central retinal thickness.
