# Supplementary material for: Double-Masked, Randomized, Phase 2 Evaluation of Abicipar Pegol (an Anti-VEGF DARPin Therapeutic) in Neovascular Age-Related Macular Degeneration
Source: J Ocul Pharmacol Ther. 2018 Dec 6;34(10):700–9. doi: 10.1089/jop.2018.0062 (PMC6306670; doi:10.1089/jop.2018.0062)
Supplement: Supplemental data [file Supp_Table3.pdf]

Supplementary Table S3. Best-Corrected Visual Acuity and Central Retinal Thickness Changes from Baseline in REACH Stage 3

Visit	*Abicipar 1 mg*	*Abicipar 2 mg*	*Ranibizumab 0.5 mg*
BCVA mean ± SD change from baseline, letters			
Week 1	2.6 ± 6.5 (n = 25)	4.6 ± 6.3 (n = 23)	2.1 ± 5.7 (n = 16)
Week 4	4.6 ± 6.0 (n = 25)	5.0 ± 7.4 (n = 23)	3.9 ± 6.0 (n = 16)
Week 8	5.9 ± 6.4 (n = 25)	9.1 ± 7.1 (n = 22)	4.1 ± 7.9 (n = 15)
Week 12	6.5 ± 6.8 (n = 24)	9.5 ± 7.4 (n = 22)	5.3 ± 10.2 (n = 16)
Week 16	5.1 ± 8.0 (n = 19)	7.6 ± 5.1 (n = 19)	5.3 ± 11.1 (n = 16)
Week 20	8.5 ± 8.1 (n = 14)	8.9 ± 5.5 (n = 13)	6.7 ± 7.7 (n = 14)
CRT mean ± SD change from baseline, μm			
Week 1	−122.9 ± 97.0 (n = 25)	−87.5 ± 61.9 (n = 23)	−78.4 ± 55.9 (n = 16)
Week 4	−168.3 ± 137.1 (n = 25)	−119.8 ± 68.5 (n = 23)	−98.4 ± 65.2 (n = 16)
Week 8	−180.3 ± 132.9 (n = 25)	−142.6 ± 78.0 (n = 22)	−109.7 ± 75.8 (n = 15)
Week 12	−183.3 ± 118.4 (n = 24)	−141.9 ± 89.8 (n = 22)	−125.8 ± 89.8 (n = 16)
Week 16	−154.0 ± 140.5 (n = 19)	−118.1 ± 97.1 (n = 19)	−125.1 ± 92.6 (n = 16)
Week 20	−86.2 ± 124.4 (n = 14)	−24.3 ± 54.1 (n = 13)	−86.1 ± 113.4 (n = 14)

Changes in BCVA and CRT from baseline are based on observed values in the modified intent-to-treat population. The number of patients in the abicipar treatment arms with observed values at weeks 16 and 20 was reduced because of data censor after escape to standard of care. Baseline mean ± SD BCVA was 58.4 ± 13.5 letters, 58.5 ± 14.3 letters, and 60.4 ± 16.4 letters in the abicipar 1 mg, abicipar 2 mg, and ranibizumab 0.5 mg arms, respectively. Baseline mean ± SD CRT was 526.1 ± 165.1 μm, 466.0 ± 126.0 μm, and 463.3 ± 94.6 μm in the abicipar 1 mg, abicipar 2 mg, and ranibizumab 0.5 mg arms, respectively.

BCVA, best-corrected visual acuity; CRT, central retinal thickness; SD, standard deviation.
