# Supplementary material for: Double-Masked, Randomized, Phase 2 Evaluation of Abicipar Pegol (an Anti-VEGF DARPin Therapeutic) in Neovascular Age-Related Macular Degeneration
Source: J Ocul Pharmacol Ther. 2018 Dec 6;34(10):700–9. doi: 10.1089/jop.2018.0062 (PMC6306670; doi:10.1089/jop.2018.0062)
Supplement: Supplemental data [file Supp_Fig3.pdf]

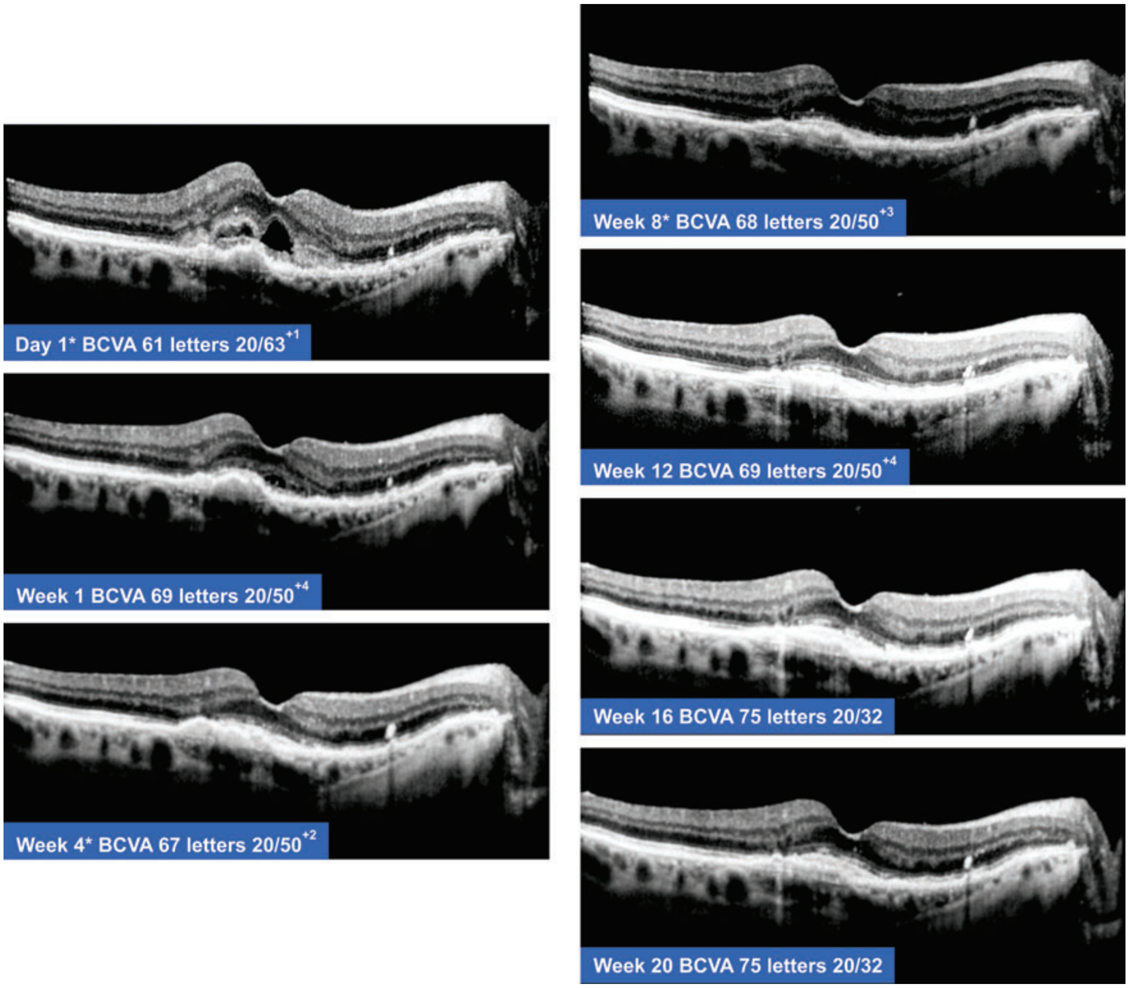

SUPPLEMENTARY FIG. S3. SD-OCT images from the study eye of a patient (a 75-year-old Hispanic woman) in the abicipar 2 mg arm with reduced visual acuity (61 letters or $20/63^{+1}$ Snellen equivalent) at baseline (day 1). Images from baseline and weeks 1, 4, 8, 12, 16, and 20 are shown with BCVA (in Early Treatment Diabetic Retinopathy Study letters) and the Snellen equivalent indicated within the *blue boxes*. Visits at which abicipar was administered (baseline, week 4, and week 8) are identified by an *asterisk*. Baseline SD-OCT images showed increased CRT with the presence of subretinal cysts. Four weeks after abicipar 2 mg treatment, retinal edema was reduced, there was complete resolution of subretinal cysts, and BCVA had improved to 67 letters. At week 16, 8 weeks after the last abicipar injection, CRT measurements indicated a reduction in CRT of ~130 μm from baseline, and BCVA had reached 75 letters. The reduction in CRT and improvement in BCVA were maintained at week 20, 12 weeks after the last abicipar injection. BCVA, best-corrected visual acuity; CRT, central retinal thickness; SD-OCT, spectral-domain optical coherence tomography.
