# Supplementary material for: Double-Masked, Randomized, Phase 2 Evaluation of Abicipar Pegol (an Anti-VEGF DARPin Therapeutic) in Neovascular Age-Related Macular Degeneration
Source: J Ocul Pharmacol Ther. 2018 Dec 6;34(10):700–9. doi: 10.1089/jop.2018.0062 (PMC6306670; doi:10.1089/jop.2018.0062)
Supplement: Supplemental data [file Supp_Fig4.pdf]

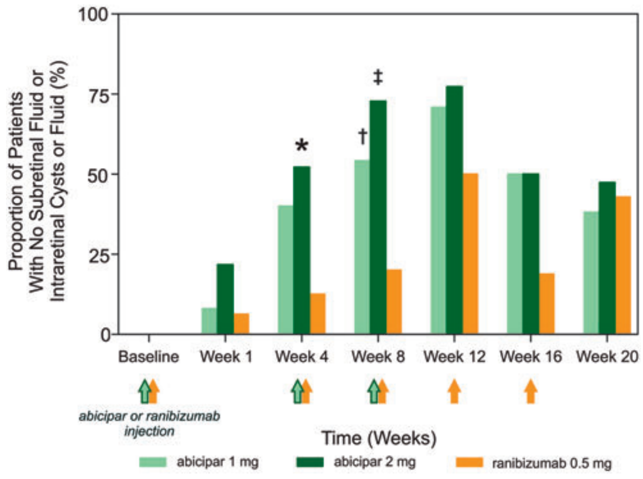

SUPPLEMENTARY FIG. S4. Proportion of patients with an ''all dry'' retinal fluid status. The analysis used all observed data, including data from patients after escape to standard of care. *Green* and *orange arrows* indicate when the 3 abicipar injections or 5 ranibizumab injections were administered. *$P = 0.017$ versus ranibizumab, †$P = 0.049$ versus ranibizumab, and ‡$P = 0.003$ versus ranibizumab.
